# Neutralization of the Principal Toxins from the Venoms of Thai *Naja kaouthia* and Malaysian *Hydrophis schistosus*: Insights into Toxin-Specific Neutralization by Two Different Antivenoms

**DOI:** 10.3390/toxins8040086

**Published:** 2016-03-26

**Authors:** Kae Yi Tan, Choo Hock Tan, Shin Yee Fung, Nget Hong Tan

**Affiliations:** 1 Department of Molecular Medicine, Faculty of Medicine, University of Malaya, Kuala Lumpur 50603, Malaysia; kytan_kae@yahoo.com (K.Y.T.); fungshinyee@gmail.com (S.Y.F.); tanngethong@yahoo.com.sg (N.H.T.); 2 Department of Pharmacology, Faculty of Medicine, University of Malaya, Kuala Lumpur 50603, Malaysia

**Keywords:** *Naja kaouthia* monovalent antivenom, sea snake antivenom, toxin-specific neutralization, antivenom potency, toxin purification

## Abstract

Antivenom neutralization against cobra venoms is generally low in potency, presumably due to poor toxin-specific immunoreactivity. This study aimed to investigate the effectiveness of two elapid antivenoms to neutralize the principal toxins purified from the venoms of the Thai monocled cobra (*Naja kaouthia*, Nk-T) and the Malaysian beaked sea snake (*Hydrophis schistosus*, Hs-M). In mice, *N. kaouthia* Monovalent Antivenom (NKMAV) neutralization against Nk-T long neurotoxin (LNTX) and cytotoxin was moderate (potency of 2.89–6.44 mg toxin/g antivenom protein) but poor against the short neurotoxin (SNTX) (1.33 mg/g). Its cross-neutralization against Hs-M LNTX of Hs-M is compatible (0.18 mg/g) but much weaker against Hs-M SNTX (0.22 mg/g). Using CSL (Seqirus Limited) Sea Snake Antivenom (SSAV), we observed consistently weak neutralization of antivenom against SNTX of both species, suggesting that this is the limiting factor on the potency of antivenom neutralization against venoms containing SNTX. Nevertheless, SSAV outperformed NKMAV in neutralizing SNTXs of both species (0.61–2.49 mg/g). The superior efficacy of SSAV against SNTX is probably partly attributable to the high abundance of SNTX in sea snake venom used as immunogen in SSAV production. The findings indicate that improving the potency of cobra antivenom may be possible with a proper immunogen formulation that seeks to overcome the limitation on SNTX immunoreactivity.

## 1. Introduction

Snakebite envenomation constitutes a highly relevant public health problem with worldwide annual mortality estimated to be at least 20,000, although the exact death figure could soar as high as 94,000 yearly [1]. Clinically, snakebite envenomation is a medical emergency needing specialized treatment in which antivenom remains the only proven and accepted antidote for use [2]. Antivenom is derived from animal immunoglobulins (commonly from horses or mules) and acts by forming immunocomplexes with toxins, thereby rendering them biologically inactive. Good-quality antivenom, when used appropriately, can effectively reduce the mortality and morbidity associated with snakebite envenomation [3]. Therefore, antivenom is included in the World Health Organization (WHO) Model List of Essential Medicine and is expected to be available in countries affected by snakebites, typically in the tropics and subtropics. However, unlike most drugs for other tropical diseases that can be genericized, the use of a particular antivenom product is very much limited to only one or several species of a certain region because of unique biogeographical distribution of snakes and diverse variations in snake venoms [3,4]. This creates a local market that is rather limited for an antivenom, accompanied by high manufacturing cost and a lack of global interest in research for the improvement of the product.

The production of antivenom with good quality and high efficacy is one of the critical steps that are needed to address the challenge of snakebite envenomation in the 21st century [5]. Antivenoms of lower efficacy are likely to be used at higher doses (involving a larger number of vials or ampoules of antivenom), leading to higher medical expenses and faster exhaustion of antivenom stock in the hospital. In addition, patients are also exposed to a larger amount of foreign proteins (antivenom) and thus at a greater risk of hypersensitive reactions that can be fatal in the worst-case scenario [6]. Therefore, studies on antivenom efficacy are essential to elucidate the neutralization profile of antivenom, and to shed light on how various limitations can be overcome for the production of a more effective antivenom. Earlier, it was shown that the neutralization potency values (*P*) of different commercial as well as experimental antivenoms toward the elapid venoms are consistently low, being in the range of less than 1 to 2 mg/mL, while values for viperid venoms are generally higher, up to 10 mg/mL [7,8]. The limitation of elapid antivenom efficacy has been suggested to be due to the presence of abundant low molecular mass toxins (in venom) that are poorly immunogenic, resulting in a low titer of effective anti-toxins. This was supported by a recent study that reported the low potency of a polyvalent antivenom (Neuro Polyvalent Antivenom) against the low molecular mass cobra neurotoxins and cytotoxins [9]. Considering that the amount of cobra venom per bite is huge (up to a few hundred milligrams), cobra antivenoms available in this region are usually required in high doses for effective neutralization. From the clinical standpoint, this is well reflected in the recommendation or practice guideline of cobra antivenom use in the region, where the initial dose of antivenom for cobra envenomation is typically a total of 10 vials of either monospecific or polyspecific antivenom [2].

On the other hand, it has been proposed that in order to cover a large range of snakes, antivenom use can be optimized according to specific toxin groups based on the principle of cross-neutralization [10,11]. The development of toxin-specific antivenom is also promising to provide better neutralization capacity by acting against the principal toxic effect of venom [10,12]. In this regard, investigating antivenom neutralization against individual toxins becomes indispensable to gain insight into the toxin-specific effectiveness of antivenom and understand how limitations can be overcome in antivenom production. In this study, the effectiveness of neutralization against the principal toxins in the venoms of the monocled cobra (*Naja kaouthia*, Thailand) and the beaked sea snake (*Hydrophis schistosus*, Malaysia) by *N. kaouthia* Monovalent Antivenom (NKMAV) and CSL Sea Snake Antivenom (SSAV) were investigated and compared. The venom of *N. kaouthia* (Thailand) contains a relatively higher amount of long neurotoxins (LNTX, up to 40%) but a moderate amount of short neurotoxins (SNTX) [13], in contrast to *H. schistosus* venom, which is predominated by SNTX (up to 60%) but moderate in LNTX content in its venom proteome [14]. Hence, the two antivenoms could potentially demonstrate meaningful variations in their immunoreactivity and neutralization profiles against the purified toxins. It is hypothesized that SSAV is more effective than NKMAV in neutralizing SNTX in addition to toxic phospholipase A_2_ (PLA_2_, also present abundantly in the immunogen for SSAV), regardless of the species origin of the toxins. It is hoped that the findings will provide deeper understanding on the toxin specificity of antivenom neutralization, thereby contributing to the improvement of manufacturing and the use of antivenom in the region.

## 2. Results

### 2.1. Isolation of Major Toxins from the Venom of Naja kaouthia

**Figure 1:**
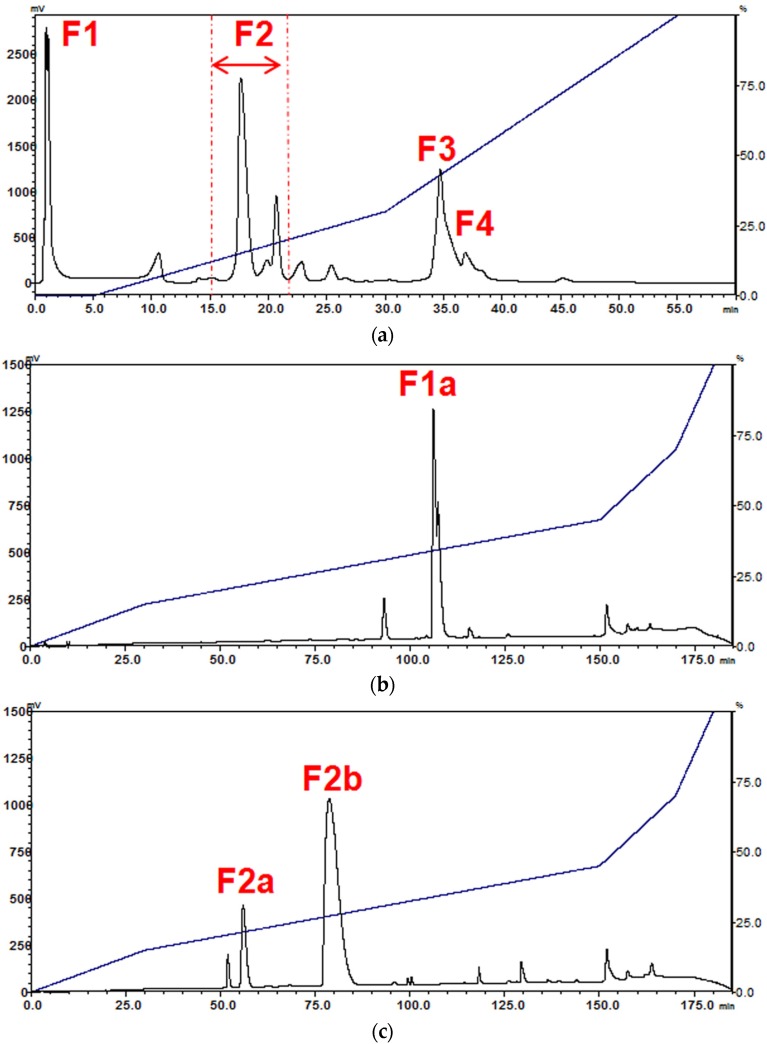

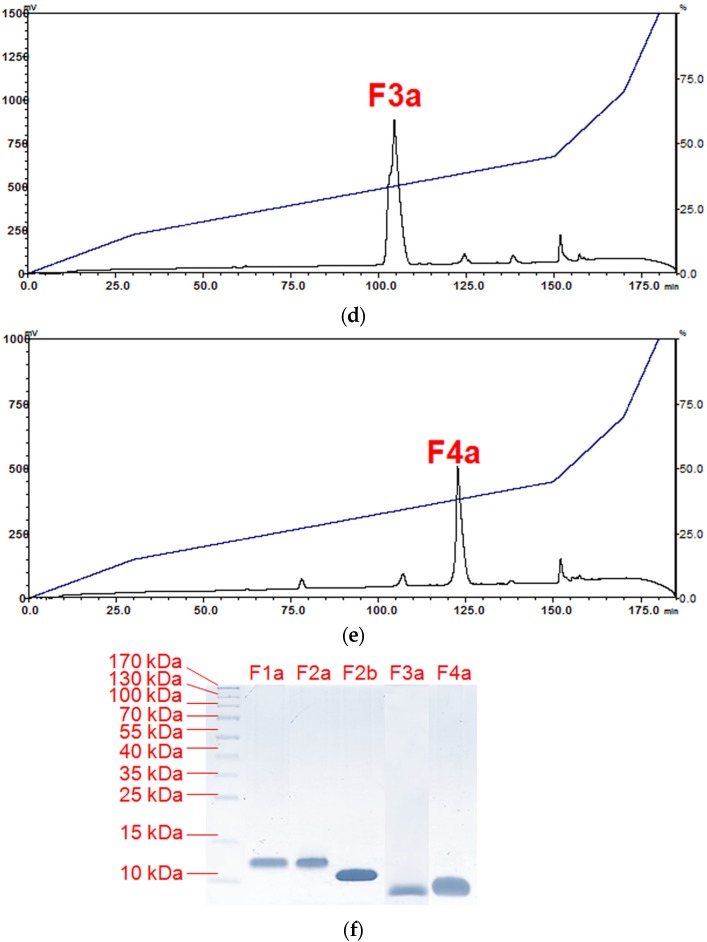
Purification of major toxins from the venom of Thai *Naja kaouthia* through sequential high performance liquid chromatography (HPLC) fractionation. (**a**) Resource S cation-exchange HPLC of 5 mg Thai *N. kaouthia* venom. Concentrated venom fractions were subjected to C_18_ RP-HPLC for further purification: (**b**) Fraction F1; (**c**) Fraction F2; (**d**) Fraction F3; (**e**) Fraction F4; (**f**) reducing SDS-PAGE of the purified venom toxins.

The venom of Thai *Naja kaouthia* (Nk-T) was resolved by cation-exchange chromatography into eight peaks, five of which constituted the majority of proteins in the venom (approximately 85% of the total peak area of the chromatogram) (Figure 1a). The five major peaks were assigned as fractions F1, F2, F3, and F4 as indicated in Figure 1a. Fractions F1–F4 were collected accordingly and were further fractionated individually by RP-HPLC over 180 min (Figure 1b–e). Fraction F1 contained acidic proteins unbound by the cation-exchange column. It was resolved into one major peak (F1a) at 105 min of reverse-phase high performance liquid chromatography (RP-HPLC). Fraction F2, the most abundant fraction comprising >35% of the total peak area of Resource S chromatogram, was separated by RP-HPLC into two main fractions (F2a and F2b) eluted at 55 min and 75 min, respectively. On cation-exchange HPLC, more basic proteins were collected in fractions F3 and F4. Although the separation of F3 and F4 appeared to be less distinctive, RP-HPLC successfully resolved each of these into one major peak, labeled as F3a (eluted at 100 min) and F4a (eluted at 120 min), respectively.

The purity of proteins in fractions F1a to F4a was verified on reducing SDS-PAGE as a single homogenous band each (7–14 kDa), indicating the unlikely presence of contaminant. The purified proteins were identified and validated using Q-TOF LCMS/MS. All proteins obtained were homologous to the proteins previously identified from the cobra genus *Naja* (Table 1). F1a is an acidic phospholipase A_2_ (PLA_2_), F2a is a short α-neurotoxin (SNTX), whereas F2b is a long α-neurotoxin (LNTX). On the other hand, F3a and F4a are two different cytotoxin (CTX) homologues, as indicated by the difference in their elution times as well as peptide sequences matched from LCMS/MS.

Together, these five purified toxins account for about 75% of the total abundance of venom proteins. The acidic PLA_2_ (F1a), termed Nk-T PLA_2_, constitutes ~17.0% of the total protein abundance. The two α-neurotoxins, SNTX (F2a) and LNTX (F2b), were named Nk-T SNTX and Nk-T LNTX, respectively, each accounting for 4.6% and 30.9% of the total abundance. The CTX homologues, labeled as Nk-T CTX-I (F3a) and Nk-T CTX-II (F4a), share 19.3% and 4.6% of total abundance, respectively (Table 2).

### 2.2. Isolation of Major Toxins from the Venom of Hydrophis schistosus

**Figure 2:**
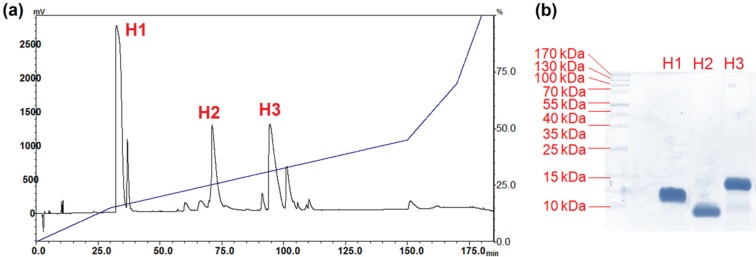
Fractionation of *Hydrophis schistosus* venom using C_18_ reverse-phase high performance liquid chromatography (RP-HPLC). (**a**) C_18_ RP-HPLC of 3 mg venom; (**b**) reducing SDS-PAGE of the purified venom toxins.

The RP-HPLC profile of *H. schistosus* venom for isolation of toxins is nearly identical to that established earlier [14]. The three toxins of interest were purified and assigned as H1, H2, and H3 in this study (Figure 2). The purity of these fractions was verified on reducing SDS-PAGE, revealing a single homogenous band each (7–14 kDa) (Figure 2). Fraction H1 is a short α-neurotoxin, the most abundant protein in *H. schistosus* venom (52.5% total venom abundance), and termed Hs-M SNTX. Fraction H2 is a long α-neurotoxin (LNTX, 11.9% of total protein abundance) labeled as Hs-M LNTX. Fraction H3 is a basic PLA_2_ (19.2% of total protein abundance) labeled as Hs-M basic PLA_2_ (Table 2). The protein abundances, intravenous median lethal doses (*i.v.* LD_50_), and toxicity scores are shown in Table 2.

### 2.3. Protein Concentration of Antivenom and Neutralization of Lethality

The concentration of SSAV is approximately five times higher than that of NKMAV. NKMAV is more effective in neutralizing Nk-T venom compared to SSAV, by comparing the values of antivenom potency (*P*) normalized by protein content (Table 3).

### 2.4. Lethality Profile and Toxicity Scoring of Purified Toxins Isolated from the Venoms of Naja kaouthia (Thailand) and Hydrophis schistosus (Malaysia)

Alpha-neurotoxins Nk-T LNTX and Nk-T SNTX are highly lethal with LD_50_ values of 0.09 and 0.12 µg/g, respectively. The cytotoxins (Nk-T CTX-I, Nk-T CTX-II) demonstrate much higher LD_50_ values of 1.41 and 1.75 µg/g. In contrast to the NTXs and CTXs, the Nk-T acidic PLA_2_ was non-lethal in mice up to 5 µg/g, a dose that is ~25 times the LD_50_ of the crude venom (0.18 µg/g). In general, the toxicity scores (TS) of most of the toxins purified from Nk-T and Hs-M venoms exceeded 5, a threshold value proposed for significant toxicity [16]. TS values for the Nk-T acidic PLA_2_ (<5) and Nk-T CTX-II (3) were exceptionally low due to their high LD_50_ values (less lethal) and/or low abundance in the venom (Table 2).

### 2.5. Antivenom Neutralization of Purified Toxins

The results of neutralization of toxins by NKMAV and SSAV were shown in Table 4. For comparison purpose, antivenom potency (*P*) was referred to the normalized value in mg/g unit (amount of venom per amount of antivenom protein). The *P* values of NKMAV were consistently low, especially against the short neurotoxins (Nk-T SNTX, *P* = 1.33 mg/g; Hs-M SNTX, *P* = 0.22 mg/g) and basic PLA_2_ (Hs-M basic PLA_2_) (*P* = 0.22 mg/g). NKMAV, however, neutralized the long neurotoxins of both species with a smaller discrepancy in the *P* values (Nk-T LNTX, *P* = 4.89 mg/g; Hs-M LNTX, 4.00 mg/g). Compared to NKMAV, SSAV was approximately twice as potent in neutralizing the Nk-T SNTX (*P* = 2.49 mg/g), but less effective against the Nk-T LNTX (*P* = 2.49 mg/g). The neutralization effect of SSAV against CTXs was negligible, in contrast with NKMAV, which was able to neutralize both Nk-T CTX-I and Nk-T CTX-II effectively with *P* values of 6.44 and 2.89 mg/g, respectively.

## 3. Discussion

Cobra venoms are highly toxic due to the effects of rapid neuromuscular paralysis and extensive tissue necrosis [2,17]. These complications are mainly attributed to the two largest protein families, *i.e.*, three-finger toxins (3FTxs) and phospholipase A_2_s (PLA_2_s), which are the major constituents of most cobra venoms [18]. The current study demonstrated the correlation between the high abundance of alpha-neurotoxins (3FTx family) and the lethality of the Thai *N. kaouthia* venom, supported by the high toxicity score (TS) values of LNTX and SNTX in the venom. In general, alpha-neurotoxins are post-synaptic nicotinic blockers that are responsible for the rapid onset of flaccid paralysis clinically [19]. Cytotoxins or cardiotoxins (CTXs) are the other 3FTx subtypes abundantly present in Nk-T venom (>20%). These toxins are less lethal when given intravenously (with higher LD_50_ and lower TS) but they play a major role in local tissue destruction (necrosis) attributable to their *in situ* cytolytic activities [20,21]. On the other hand, the major phospholipase A_2_ isolated from Nk-T venom is an acidic isoform that exhibits negligible lethality when given intravenously to mice, consistent with the findings of non-toxic acidic PLA_2_ from *N. kaouthia* venom of Indian origin [22]. The potentially toxic role of the acidic PLA_2_ remains to be further investigated. Of note, lethal neutral PLA_2_ has been reported from the venom of the equatorial spitting cobra (*Naja sumatrana*, Malaysia) [9], while myotoxic basic PLA_2_s, although rarely reported for cobra venoms, are important lethal principles of venoms of some elapids such as sea snakes [14]. In view of its potent lethality and high abundance, the basic PLA_2_ of *H. schistosus* (Hs-M basic PLA_2_) also exhibits a high toxicity score comparable to those of the long and short neurotoxins in the venom. It should be noted that although toxicity scoring can serve as an indicator to gauge the significance of a toxin in contributing to venom lethality [16], the usefulness of this approach is generally confined to elapid venoms, particularly those dominated by low molecular mass toxins. Viperid or crotalid venoms, on the contrary, are composed of various proteins of moderate to high molecular mass that generally have higher LD_50_ but act in synergy. Hence, for viperid and crotalid venoms, the toxicity scoring system is unlikely to reflect the true contribution of an individual toxin to venom lethality.

The capacity of two antivenoms to neutralize the major toxins of *N. kaouthia* and *H. schistosus* venoms was further examined and compared with their capacity to neutralize the whole venom. Earlier, cobra antivenoms were reported to confer cross-neutralization against sea snake venom [15], although the potency of neutralization is rather low (*P* = 0.03 mg/mL, or 0.67 mg/g) in comparison with its neutralization potency against the homologous Nk-T venom (*P* = 0.92 mg/mL, or 20.44 mg/g) [13]. In this study, the ability of CSL Sea Snake Antivenom (SSAV) to effectively cross-neutralize *N. kaouthia* venom is therefore unsurprising and supports the fact that common toxin antigens or immunological determinants are shared between the cobra and sea snake venoms, as also suggested by a few earlier studies [15,23,24]. Of note, both *N. kaouthia* and *H. schistosus* venoms act primarily by neuromuscular paralysis, attributable to the high content of alpha-neurotoxins in both venoms, which likely exhibit similar epitopes. In addition, the cross-reactivity of SSAV with cobra venom might be also contributed by the inclusion of the Australian tiger snake venom in the sea snake venom immunogen during antivenom production. Earlier, O’Leary *et al.* [25] showed that horses used in the production of some CSL antivenoms (for instance, the Australian brown snake antivenom and tiger snake antivenom) were injected with multiple snake venoms. Where SSAV is concerned, this antivenom is known as a bivalent product raised against the venoms of *H. schistosus* and *N. scutatus*, and the efficacy of this antivenom against the *in vitro* neurotoxic effect of the two venoms (from sea snake and tiger snake) has been previously examined, with the findings indicating that SSAV is more effective against sea snakes compared to *N. scutatus* [26]. Nonetheless, commercial Australian tiger snake antivenom was shown to be able to cross-neutralize the neurotoxic effect of Egyptian cobra venom in mice, as reported by Kornhauser [10]. These reports support the idea that *N. scutatus* venom, which contains abundant PLA_2_, might play a role in enhancing the potency of SSAV, especially in cross-neutralizing the toxic PLA_2_ of sea snake, as observed in this study.

The composition of neurotoxin subtypes, *i.e.*, short neurotoxins (SNTX) and long neurotoxins (LNTX), is known to be quite different between the venoms of Thai *N. kaouthia* (Nk-T) and Malaysian *H. schistosus* (Hs-M). Nk-T venom is predominated by LNTX (33.3%) and has less SNTX (7.7%) [13], while Hs-M venom is predominated by SNTX (55.8%) and has less LNTX (14.7%) [14]. The lack of SNTX in Nk-T venom may be the reason for NKMAV consistently exhibiting low neutralization potency against the SNTXs of homologous (Nk-T SNTX, *P* = 1.33 mg/g) and heterologous (Hs-M SNTX, *P* = 2.49 mg/g) origins. This observation is in agreement with previous reports on low immunoreactivity and the weak neutralization effect of cobra antivenom against neurotoxins of short-chain isoforms [9,15,27], and indicates that short neurotoxins of different species origin can vary in subtle structures, resulting in alteration of protein antigenicity. In addition, the poor cross-neutralization of Hs-M basic PLA_2_ by NKMAV correlates with the absence of basic PLA_2_ in the immunization process to produce NKMAV. Nonetheless, the small discrepancy between the neutralization potencies of NKMAV against the LNTX of both species suggests that the antigenic sites of the Hs-M LNTX are likely conserved between the two lineages.

On the other hand, SSAV neutralized Hs-M LNTX and Hs-M basic PLA_2_ effectively (*P* value up to 6.35 mg/g) but was less potent against its Hs-M SNTX (*P* = 2.49 mg/mL), indicating that SNTX is a relatively poor immunogen among the few toxins in the venom. Nevertheless, SSAV still outperformed NKMAV in neutralizing the short neurotoxins of both species (Nk-T SNTX and Hs-M SNTX) several fold over in term of potency. The higher immunoreactivity and neutralizing capacity of SSAV against SNTX suggest the possibility of overcoming the limitation of cobra antivenom in neutralizing venoms that contain abundant SNTX. SSAV neutralization against the long neurotoxin of *N. kaouthia* (Nk-T LNTX) is, however, less effective compared to neutralization by the homologous NKMAV, indicating that Nk-T LNTX may exhibit unique epitopes not shared by Hs-M LNTX. This may also contribute to the lower potency of SSAV in neutralizing Nk-T venom, which predominantly contains LNTXs (>30% of total venom proteins) as its lethalprinciple. It is also known that the immunogen mix of SSAV consists of primarily SNTX, LNTX, and basic PLA_2_ while lacking cobra cytotoxins (CTX); therefore, it is not surprising that SSAV was ineffective against the two CTXs of *N. kaouthia* (*P* = 0.00–0.41 mg/g). Moreover, it is important to note that the low neutralization potency against the CTXs (Nk-T CTX-I and Nk-T CTX-II, apart from SNTX) by NKMAV indicates that weak neutralization against CTX may further restrict the potency and efficacy of a cobra antivenom [9], although in general CTXs show lower toxicity scores due to their higher LD_50_ values. Often, the effect of CTX is implicated in local tissue destruction, which leads to crippling disability in the survivors, while the role of antivenom in neutralizing the local effect of venom has not been very well established. Meanwhile, SSAV was much more potent than NKMAV against the toxic (basic) PLA_2_ of *H. schistosus* venom, consistent with the substantial presence of this enzyme in SSAV immunogen (*H. schistosus* and *N. scutatus* venoms). *Naja kaouthia* venom, used for the production of NKMAV, lacks toxic (basic) PLA_2_ [13].

Taken together, the current study showed that the relatively poor immunogenicity of the low molecular mass toxins, especially SNTX, is the main limiting factor on the efficacy of cobra antivenom. Also, we demonstrated that SSAV exhibited higher potency in neutralizing the lethal effect of short neurotoxins and lethal PLA_2_ compared to NKMAV. It is likely possible that the higher immunoreactivity of SSAV toward short neurotoxins is attributed at least partly to the high SNTX content of the sea snake venom [14] used as immunogen in the production of SSAV. Other factors such as the intrinsic structure of each toxin may also contribute to variations in venom immunogenicity.

## 4. Conclusions

The study reveals the possibility of improving the potency of cobra antivenom whereby the antivenom production should aim to overcome the limitation of neutralization against alpha-neurotoxins, particularly the short NTX subtype. In addition to the low dose, low volume, multi-site immunization protocol [28,29] that has been widely practiced, it is necessary to look into how the immunogen formulation can be further optimized. A proper immunogen mix, which includes various essential low molecular mass toxins in a sufficient amount (toxin-enriched immunogen), with or without cross-linking approach of small polypeptides [30,31], should be developed in order to enhance the immunogenicity of the toxins and to increase the anti-toxin titers. Coupled with recent understandings on venomics and quantitative expression of toxins [13,32], it is hoped that *in vivo* neutralization assessment of individual toxins will provide deeper insights into the immunoreactivity of antivenom, thereby improving the efficacy and quality of antivenom product in the region.

## 5. Materials and Methods

### 5.1. Venoms and Antivenoms

The pooled venom of Thai *Naja kaouthia* (Nk-T) was a generous gift from Professor Kavi Ratanabanangkoon of the Chulabhorn Graduate Institute, Bangkok, Thailand. *Hydrophis schistosus* venom was collected and pooled from 20 adult specimens in the northwestern waters of Peninsular Malaya (State of Penang). All venoms were in lyophilized form and stored at −20 °C until use. The antivenoms used were as follows: (a) *Naja kaouthia* Monovalent Antivenom (NKMAV, produced by Queen Saovabha Memorial Institute (QSMI), Bangkok, Thailand; batch: 0080210; expiry date: 9 August 2015) that contains purified F(ab′)_2_ derived from horse IgG, indicated as the treatment of *N. kaouthia* envenoming; (b) Australian CSL Sea Snake Antivenom (SSAV, produced by CSL Limited, Parkville, Victoria, Australia, recently operates under the brand *Seqirus*; batch: 0549-08201; expiry date: April 2015). SSAV contains purified F(ab′)_2_ derived from horses immunized with the venoms of *Hydrophis schistosus* (formerly *Enhydrina schistosa*) and the Australian tiger snake (*Notechis scutatus*) and is indicated as the treatment for envenoming by *Hydrophis schistosus* and other sea snakes. NKMAV (lyophilized) was reconstituted according to the attached product leaflet: 10 mL of normal saline was added to one vial of the lyophilized antivenom. According to the product leaflet, 1 mL of NKMAV antivenom is able to neutralize 0.6 mg of *N. kaouthia* venom. SSAV used in this study was packaged as 25 mL liquid in an ampoule containing 1000 units of neutralizing capacity against the target venom of *H. schistosus*.

### 5.2. Animals and Ethics Clearance

Mice used in this study were of albino ICR strain (20–25 g) supplied by the Animal Experimental Unit, University of Malaya. The protocol of animal studies was based on the Council for International Organizations of Medical Sciences (CIOMS) guidelines [33] on animal experimentation and was approved by the Institutional Animal Care and Use Committee of the University of Malaya (Ethics clearance number: 2014-09-11/PHAR/R/TCH, date of approval: 11 September 2014).

### 5.3. Chemicals and Materials

All chemicals and reagents used were of analytical grade. Ammonium bicarbonate, dithiothreitol (DTT) and iodoacetamide were purchased from Sigma-Aldrich (St. Louis, MO, USA). MS grade trypsin protease, Spectra™ Multicolor Broad Range Protein Ladder (10 to 170 kDa), and HPLC grade solvents used in the studies were purchased from Thermo^®^ Scientific Pierce (Waltham, MA, USA). Resource^®^ S cation-exchange column (1 mL) was purchased from GE Healthcare (Uppsala, Sweden). LiChroCART^®^ 250-4 LiChrospher^®^ WP 300 and Millipore ZipTip^®^ C_18_ Pipette Tips were purchased from Merck (Kenilworth, NJ, USA).

### 5.4. Estimation of Protein Concentration in Antivenom

Protein concentrations in antivenoms (NKMAV and CSL SSAV) were determined using Thermo Scientific™ Pierce™ BCA (bicinchoninic acid) Protein Assay Kit. The protein concentrations were expressed as means ± SEM of triplicates.

### 5.5. Isolation and Purification of Major Toxins from the Venom of Naja kaouthia and Hydrophis schistosus

The isolation and purification of the major toxins from *Naja kaouthia* venom were conducted as described previously [9] through sequential fractionation using Resource^®^ S cation-exchange liquid chromatography followed by reverse-phase high performance liquid chromatography (RP-HPLC) on a Shimadzu LC-20AD High Performance Liquid Chromatography system (Shimadzu, Kyoto, Japan). The isolation of toxins from *Hydrophis schistosus* venom was carried out according to the protocol on reverse-phase high performance liquid chromatography (RP-HPLC) reported recently [14].

#### 5.5.1. Fractionation of *N. kaouthia* Venom Using Resource S Ion-Exchange Chromatography

Venom was reconstituted and subjected to cation-exchange chromatography using a Resource^®^ S column pre-equilibrated with 20 mM 2-(*N*-morpholino)ethanesulfonic acid (MES), pH 6.0 as eluent A. Elution was achieved with 0.8 M NaCl in 20 mM MES, pH 6.0 as eluent B, using a linear gradient flow of 0%–30% B from 5 to 40 min followed by 30%–100% B from 40 to 55 min. The flow rate was set at 1.0 mL/min and the venom fractions were collected manually at absorbance detection of 280 nm.

#### 5.5.2. Purification of Venom Toxins Using Reverse-Phase HPLC

The protein fractions collected from cation-exchange chromatography were concentrated and subjected to further purification by reverse-phase high performance liquid chromatography (RP-HPLC) with a C_18_ reverse-phase column (5 µm). The fractions were eluted at 1 mL/min with a linear gradient of 0.1% trifluoroacetic acid (TFA) in water (Solvent A) and 0.1% of TFA in acetonitrile (Solvent B) (0%–5% B for 10 min, 5%–15% B over 20 min, 15%–45% B over 120 min and 45%–70% B over 20 min). Purified toxins were detected at absorbance 215 nm, collected manually, subsequently lyophilized, and stored at −20 °C until use.

### 5.6. SDS-PAGE and In-Solution Tryptic Digestion of Purified Toxins

The purified toxins were subjected to 15% sodium dodecyl sulphate polyacrylamide gel electrophoresis (SDS-PAGE) under reducing condition as described by Laemmli [34] and calibrated with the Thermo™ Scientific™ PageRuler Prestained Protein Ladder (10–170 kDa). Separately, the purified toxins were subjected to reduction with DTT, alkylation with iodoacetamide, and in-solution digestion with mass-spectrometry grade trypsin protease according to the manufacturer’s protocol (Thermo Scientific™ Pierce™). The trypsin-digested peptides were desalted with Millipore ZipTip^®^ C_18_ Pipette Tips (Merck) according to the manufacturer’s protocol to enhance the performance of mass spectrometry.

### 5.7. Protein Identification by Liquid Chromatography-Tandem Mass Spectrometry

Samples were loaded in a large capacity chip 300 Å, C18, 160 nL enrichment column and 75 μm × 150 mm analytical column (Agilent part No. G4240-62010) with a flow rate of 4 μL/min from a capillary pump and 0.4 μL/min from a Nano pump of Agilent 1200 series. The digested peptide eluates were then subjected to nano-electrospray ionization (ESI) MS/MS experiments using an Agilent 1200 HPLC-Chip/MS Interface, coupled with Agilent 6550 Accurate-Mass Q-TOF LC/MS system. Injection volume was adjusted to 2 μL per sample and the mobile phases were 0.1% formic acid in water (A) and 100% acetonitrile with 0.1% formic acid (B). The gradient applied was: 5%–50% solution B for 11 min, 50%–70% solution B for 4 min, and 70% solution B for 3 min, using Agilent 1200 series nano-flow LC pump. Ion polarity was set to positive ionization mode. Drying gas flow rate was 11 L/min and drying gas temperature was 290 °C. Fragmentor voltage was 175 V and the capillary voltage was set to 1800 V. Spectra were acquired in a MS/MS mode with an MS scan range of 200–3000 *m*/*z* and MS/MS scan range of 50–3200 *m*/*z*. Precursor charge selection was set as doubly charged state and above with the exclusion of precursors 1221.9906 *m*/*z* (*z* = 1) and 299.2944 (*z* = 1) set as reference ions. Data were extracted with MH^+^ mass range between 50 and 3200 Da and processed with Agilent Spectrum Mill MS Proteomics Workbench software packages (Version B.04.00, Agilent Technologies, Santa Clara, USA, 2012). Carbamidomethylation of cysteine was set as a fixed modification. The peptide finger mapping was modified to specifically search against non-redundant NCBI database with taxonomy set to Serpentes (taxid: 8570). Protein identifications were validated with the following filters: protein score > 20, peptides score > 6 and scored peak intensity (SPI) > 70%. False discovery rate (FDR) was less than 1% for peptides and 0% for protein identification.

### 5.8. Estimation of the Relative Protein Abundance of Purified Toxin

The relative abundance of individual protein fraction from ion-exchange or reverse-phase HPLC was estimated based on the peak area measurement using Shimadzu LCsolution Software (Version 1.23, Shimadzu, Kyoto, Japan, 2007). The total area under the curve for each chromatogram (ion-exchange or reverse-phase) was represented as 100%. The final abundance of a purified toxin was the product of multiplying the percentage of the peak areas implicated based on its sequential elution profiles involving the two chromatographic columns.

### 5.9. Determination of Lethality of Venoms and Their Purified Toxins

The determination of intravenous (*i.v.*) median lethal dose (LD_50_) for venoms or toxins was performed in mice according to a recent *in vivo* assay [14]. The venoms or toxins were injected intravenously (via the tail caudal vein) into albino mice of ICR strain (20–25 g) at appropriate doses (*n* = 4 per dose). The mice were allowed *ad libitum* access to food and water, and the survival ratio of mice at each dose was recorded at 48 h post-injection. Median lethal doses (LD_50_) were estimated using the Probit analysis method [35]. Toxicity Score of purified toxins was estimated as previously defined [16], representing the ratio of toxin abundance (%) to its LD_50_ value.

### 5.10. Neutralization of Venoms and Purified Toxins in a Mouse Model

This was adapted from the venom/toxin-antivenom immunocomplexation method reported in mice [13]. Venom or toxin at a challenge dose of 5× LD_50_ was mixed with antivenom of different concentrations to give a total volume of 200 µL. The mixtures were pre-incubated at 37 °C for 30 min and then injected intravenously into the mice (*n* = 4 per dose). The mice were allowed *ad libitum* access to food and water, and the ratio of survival was recorded at 24 h post injection. Neutralizing capacity was expressed as ED_50_ defined as the amount of reconstituted antivenom that gives 50% survival in the venom-challenged animals. If 200 µL of reconstituted antivenom failed to give the mice full protection, a lower challenge dose (2.5× or 1.5× LD_50_) was used. All challenge doses were proven to be above 100% lethal dose (LD_100_) when given intravenously, as observed in the LD_50_ determination assay as well as in the neutralization (quantal dose-response) study where lethality occurred at lower doses of antivenom, used dose-dependently. In addition, based on the total amount of venom or toxin injected, ER_50_ values were calculated (ER_50_, median effective ratio, was defined as the ratio of the amount of venom (mg) to the volume dose of antivenom (mL) to give 50% survival in the mice challenged). Neutralization capacity was also expressed in term of “neutralization potency” (*P*, defined as the amount of venom neutralized completely by a unit of antivenom) according to the calculation of Morais *et al.* [36]. The neutralization potency is a more direct indicator of antivenom neutralizing capacity, and is theoretically unaffected by the number of LD_50_ in the challenge dose. For comparative purposes, *P* values of different antivenoms were normalized by their respective protein amount and expressed as milligram of venom neutralized per gram of antivenom protein.

## Figures and Tables

**Table 1:** Protein identification of the toxins purified from *Naja kaouthia* (Thailand) venom by nano-ESI-LCMS/MS and their respective protein abundances.

Protein Fraction	%	Protein ID	MS/MS Derived Sequence	Matched Peptide	Matched MH^+^	MH^+^ Error (ppm)	Accession No. (Species)	Protein Score
F1a	17.0	Acidic PLA_2_ 1	CCQVHDNCYNEAEK	1	1828.70	−0.3	P00596 (*N. kaouthia*)	187
			CCQVHDNCYNEAEK	1	1827.69	−2.1		
			CWPYFK	1	901.42	0.9		
			CWPYFKTYSYECSQGTLTCK	1	2581.12	−1.0		
			CWPYFKTYSYECSQGTLTCK	1	2580.11	0.0		
			GDNDACAAAVCDCDR	1	1670.61	0.5		
			GDNDACAAAVCDCDR	1	1671.62	1.0		
			LAAICFAGAPYNNNNYNIDLK	3	2357.14	−1.3		
			LAAICFAGAPYNNNNYNIDLK	1	2358.15	−1.4		
			LAAICFAGAPYNNNNYNIDLK	4	2358.15	0.4		
			LAAICFAGAPYNNNNYNIDLK	1	2358.15	−0.6		
			LAAICFAGAPYNNNNYNIDLK	1	2359.16	1.0		
			LAAICFAGAPYNNNNYNIDLKAR	1	2585.29	−0.2		
			NMIQCTVPNR	1	1249.59	−0.8		
			NMIQCTVPNR	1	1234.61	5.4		
			SWWDFADYGCYCGR	1	1843.71	0.6		
			SWWDFADYGCYCGR	2	1844.72	0.5		
			SWWDFADYGCYCGR	1	1843.71	−0.6		
			SWWDFADYGCYCGR	1	1843.71	−0.4		
			TYSYECSQGTLTCK	1	1698.72	0.6		
			TYSYECSQGTLTCK	1	1699.73	2.2		
			TYSYECSQGTLTCK	1	1698.72	0.1		
F2a	4.6	Cobrotoxin-c	LECHNQQSSQAPTTK	1	1730.81	1.1	P59276 (*N. kaouthia*)	64
			LECHNQQSSQAPTTKTCSGETNCYK	1	2932.27	−1.9		
			LECHNQQSSQAPTTKTCSGETNCYK	1	2931.26	−2.1		
			VKPGVNLNCCR	1	1317.66	0.6		
F2b	30.9	Alpha-elapitoxin Nk2a	CFITPDITSK	5	1182.60	1.6	P01391 (*N. kaouthia*)	101
			RVDLGCAATCPTVK	1	1549.81	15.3		
			TGVDIQCCSTDNCNPFPTR	1	2242.94	−0.2		
			TGVDIQCCSTDNCNPFPTR	1	2243.95	2.1		
			TGVDIQCCSTDNCNPFPTR	1	2244.96	2.7		
			TGVDIQCCSTDNCNPFPTRK	1	2372.04	0.9		
			TGVDIQCCSTDNCNPFPTRK	1	2371.03	−2.0		
			TWCDAFCSIR	2	1316.57	1.5		
			TWCDAFCSIR	1	1317.57	2.7		
			TWCDAFCSIR	1	1316.56	1.0		
			VDLGCAATCPTVK	1	1393.68	2.2		
F3a	19.2	Cytotoxin 2	GCIDVCPKNSLLVK	1	1603.84	0.1	P01445 (*N. kaouthia*)	82
			LIPLAYK	1	818.53	0.6		
			LIPLAYK	5	818.53	0.9		
			LIPLAYK	1	818.53	3.8		
			LIPLAYKTCPAGK	1	1433.82	3.5		
			NSLLVKYVCCNTDR	1	1742.85	1.7		
			YVCCNTDR	1	1088.44	0.4		
F4a	4.6	Cytotoxin	CNKLVPLFYKTCPAGK	1	1897.99	−7.6	Q02454 (*N. sputatrix*)	146
			MFMVATPKVPVK	1	1349.76	−4.5		
			LKCNKLVPLFYK	1	1523.88	−3.0		
			SSLLVKYVCCNTDR	1	1715.83	−2.2		
			YVCCNTDR	1	1088.44	−0.8		
			GCIDVCPKSSLLVK	1	1576.83	−0.5		
			NLCYKMFMVATPK	1	1603.79	0.4		
			SSLLVKYVCCNTDR	1	1716.84	0.6		
			LVPLFYKTCPAGK	1	1495.83	0.8		
			MFMVATPK	2	925.48	1.7		
			LVPLFYK	6	880.54	2.6		

**Table 2:** Intravenous median lethal doses (LD_50_) of toxins purified from *Naja kaouthia* and *Hydrophis schistosus* venoms and Toxicity Scores (TS) for toxins.

Venom	Toxin Abundance in Venom (%)	*i.v.* LD_50_ (µg/g)	Toxicity Score (g/µg)
***Naja kaouthia* (Thailand)**		0.18 (0.12–0.27) ^#^	
**F1a (Nk-T acidic PLA_2_)**	17.0	>5.00	<5
**F2a (Nk-T SNTX)**	4.6	0.12 (0.11–0.14)	38
**F2b (Nk-T LNTX)**	30.9	0.09 (0.06–0.14)	343
**F3a (Nk-T CTX-I)**	19.2	1.41 (1.08–1.85)	14
**F4a (Nk-T CTX-II)**	4.6	1.75 (1.68–1.83)	3
***Hydrophis schistosus* (Malaysia)**		0.07 (0.05–0.09) ^@^	
**H1 (Hs-M SNTX)**	52.2 *	0.07 (0.05–0.09) *	746
**H2 (Hs-M LNTX)**	11.9 *	0.18 (0.16–0.20) *	66
**H3 (Hs-M basic PLA_2_)**	19.2 *	0.08 (0.06–0.10) *	240

Reference values from the same laboratories: ^#^ [13]; ^@^ [15]; * [14]. Toxicity Score was defined as the ratio of protein abundance of a toxin (%) divided by its median lethal dose (LD_50_) [16].

**Table 3:** Protein concentrations of *N. kaouthia* Monovalent Antivenom (NKMAV) and CSL Sea Snake Antivenom (SSAV) and neutralization of *N. kaouthia* venom lethality by the antivenoms.

Antivenom	Protein Concentration (mg/mL)	Neutralization of *Naja kaouthia* Venom			
		ED_50_ (µL)	ER_50_ (mg/mL)	*P* (mg/mL)	Normalized *P* (mg/g)
NKMAV	45.0 ± 0.6	18.75 ^#^	1.15 ^#^ (0.77–1.73)	0.92 ^#^	20.44
SSAV	217.2 ± 3.0	11.24	2.00 (1.33–3.00)	1.60	7.37

Intravenous LD_50_ of *N. kaouthia* value (0.18 µg/g) and reference values ^#^ were adopted from Tan *et al.* [13]. Challenge dose in mice was formulated as 5× *i.v.* median lethal dose (LD_50_) of the venom. *i.v*.: intravenous; ED_50_: antivenom dose (µL) at which 50% of mice survived; ER_50_: median effective ratio, ratio of the amount of venom (mg) to the volume dose of antivenom (mL) at which 50% of mice survived; *P*: potency expressed as the amount of venom completely neutralized by one mL antivenom. Normalized *P* was defined as the amount of venom (mg) completely neutralized per unit amount of antivenom protein (g).

**Table 4:** Neutralization of lethality of isolated toxins by *Naja kaouthia* Monovalent Antivenom (NKMAV) and CSL Sea Snake Antivenom (SSAV).

Venom Toxin	*i.v.* LD_50_ (µg/g)	NKMAV					SSAV				
		Challenge	ED_50_ (µL)	ER_50_ (mg/mL)	*P* (mg/mL)	Normalized *P* (mg/g)	Challenge	ED_50_ (µL)	ER_50_ (mg/mL)	*P* (mg/mL)	Normalized *P* (mg/g)
***Naja kaouthia* (Thailand)**											
**F2a (Nk-T SNTX)**	0.12 (0.11–0.14)	2.5 LD_50_	70.68	0.10 (0.09–0.12)	0.06	1.33	5 LD_50_	21.37	0.67 (0.62–0.79)	0.54	2.49
**F2b (Nk-T LNTX)**	0.09 (0.06–0.14)	5 LD_50_	39.14	0.28 (0.18–0.43)	0.22	4.89	5 LD_50_	16.05	0.67 (0.45–1.05)	0.54	2.49
**F3a (Nk-T CTX-I)**	1.41 (1.08–1.85)	1.5 LD_50_	53.16	0.875 (0.670–1.148)	0.29	6.44	1.5 LD_50_	175.00	0.27 (0.20–0.35)	0.09	0.41
**F4a (Nk-T CTX-II)**	1.75 (1.68–1.83)	1.5 LD_50_	156.57	0.40 (0.39–0.42)	0.13	2.89	1.5 LD_50_	N.E.	N.E.	N.E.	N.E.
***Hydrophis schistosus* (Malaysia)**											
**F1 (Hs-M SNTX)**	0.07 (0.05–0.09) *	1.5 LD_50_	128.41	0.02 (0.01–0.02)	0.01	0.22	5 LD_50_	17.67	0.44 (0.31–0.56)	0.35	1.61
**F2 (Hs-M LNTX)**	0.18 (0.16–0.20) *	5 LD_50_	81.25	0.22 (0.20–0.25)	0.18	4.00	5 LD_50_	11.98	1.73 (1.54–1.92)	1.38	6.35
**F3 (Hs-M basic PLA_2_)**	0.08 (0.06–0.10) *	1.5 LD_50_	125.00	0.02 (0.01–0.02)	0.01	0.22	5 LD_50_	5.62	1.57 (1.17–1.96)	1.25	5.76

* Reference values from Tan *et al.* [14]. All challenge doses were proven to be above the 100% lethal dose (LD_100_) when given intravenously without antivenom. Antivenom is considered non-effective when the maximum volume (200 µL) of antivenom used in the immunocomplexation failed to protect the mice from the lethal effect of the venom at a minimum challenge dose of 1.5 LD_50_. *i.v*.: intravenous; LD_50_: median lethal dose; ED_50_: antivenom dose (µL) at which 50% of mice survived; ER_50_: median effective ratio, ratio of the amount of venom (mg) to the volume dose of antivenom (mL) at which 50% of mice survived; *P*: potency expressed as the amount of venom (mg) completely neutralized by one mL antivenom; normalized *P* was defined as the amount of toxin (mg) completely neutralized per unit amount of antivenom protein (g). N.E.: non-effective.

## Abbreviations

The following abbreviations are used in this manuscript:

Nk-T	*Naja kaouthia* of Thailand
Hs-M	*Hydrophis schistosus* of Malaysia
NKMAV	*Naja kaouthia* Monovalent Antivenom
SSAV	Sea Snake Antivenom
SNTX	Short neurotoxin
LNTX	Long neurotoxin
CTX	Cytotoxin
PLA_2_	Phospholipase A_2_
LD_50_	Median lethal dose
ED_50_	Dose at which 50% of animals survived (µL)
ER_50_	Median effective ratio, ratio of the amount of venom (mg) to the volume dose of antivenom (mL) at which 50% of mice survived
*P*	Potency
T.S.	Toxicity score
